# Supplementary figures and images for: Automatic classification of lymphoma lesions in FDG-PET–Differentiation between tumor and non-tumor uptake
Source: PLoS One. 2022 Apr 18;17(4):e0267275. doi: 10.1371/journal.pone.0267275 (PMC9015138; doi:10.1371/journal.pone.0267275)

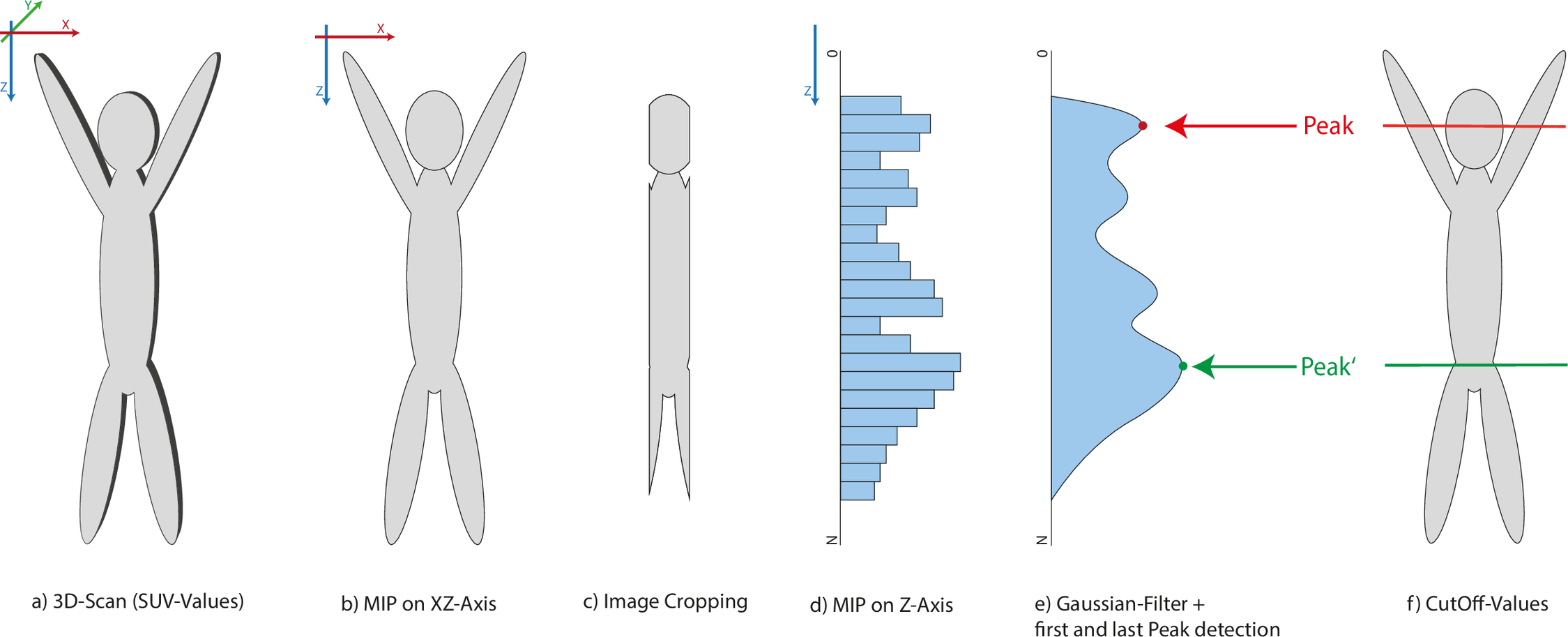

Supplement: S1 Fig — The aim of heuristic cropping was to detect the brain and the urinary bladder as the cranial and caudal reference points. In most cases both show a distinct physiological uptake and therefore are a viable targets. In a first step the 3D SUV image (a) was projected onto the xz-plane (b), cropped to exclude extremities (c), and further projected onto the z-axis to create a function of SUV maxima from cranial to caudal (d). In a seconded step we smoothed the function and applied a peak detection to extract the first and last significant peak (e). Those correlated in most cases with brain and bladder and served as boundaries for cropping. (TIF) [file pone.0267275.s001.tif]
