# Supplementary material for: Automatic classification of lymphoma lesions in FDG-PET–Differentiation between tumor and non-tumor uptake
Source: PLoS One. 2022 Apr 18;17(4):e0267275. doi: 10.1371/journal.pone.0267275 (PMC9015138; doi:10.1371/journal.pone.0267275)
Supplement: S1 File — (DOCX) [file pone.0267275.s002.docx]

**S1 File. Explanation of the nested cross validation.**

Nested cross validation was used for training of the random forest including hyperparameters. This procedure is first described for a large dataset without the use of cross validation. Nested cross validation is described in detail in the subsequent paragraph.

The whole dataset is split into three parts, a training, a validation and a test set. Using the training set, a random forest is trained with the supervised learning paradigm. All decision trees of a random forest are independently trained as follows: Starting with the complete training set, the first decision of the tree is determined as the feature and associated cut-off value which splits this set as "pure" as possible with respect to the categories. Only a subset of all features is considered, which for each decision is randomly sampled from all features. Maximum purity in this context is given, if each of the two sets consists of volumes with identical manually classified categories. The optimal decision is applied and the procedure recursively iterated for the resulting two subsets, until a maximum depth is reached or all subsets are completely pure. This training is conducted for different combinations of hyperparameters, in our study the maximum depth of each decision tree and the number of selectable features for each decision. Each resulting random forest is tested on the validation set to determine the best combination of hyperparameters. The latter is used to train another random forest based on the combined training and validation sets. The resulting random forest is applied to the test set to measure the performance.

If the available dataset is not sufficiently large to split the data into three sets, a cross validation is used.

First, the situation is considered, where no hyperparameters need to be determined. The complete dataset is randomly split into K folds. In turn each of the K folds is considered as test set. Using the remaining K-1 folds as training data, a classifier is learned and applied to the test data. Thus, each scan of the dataset is used exactly once for testing. The performance is aggregated for the K test folds.

If hyperparameters need to be optimized in addition, a nested cross validation is employed. As for the simple cross validation, the dataset is split into K outer folds. One of the K outer folds is defined as test data and the remaining K-1 folds are used for a (simple) cross validation to determine hyperparameters: The K-1 folds are once again split into K inner folds. In turn, each of these inner folds is used as a validation set. The remaining K-1 inner folds are used for training with each hyperparameter set and each resulting classifier is applied to the validation set. This is iterated for each of the inner folds as validation data. The performance measurements are aggregated and the best set of hyperparameters is selected. Using the current training and validation data, a new classifier is trained with the optimal hyperparameter set and applied to the test data, i.e. one of the K outer folds. This procedure is carried out for each of the K outer folds as test data and as for the simple cross validation the aggregated performance yields an unbiased estimate. In our experiments we used K=3 for the inner and outer folds. Note, that in general different sets of optimal hyperparameters result in the inner cross validation. In addition, the number of folds need not to be identical for the inner and outer fold.
