# Supplementary material for: Automatic classification of lymphoma lesions in FDG-PET–Differentiation between tumor and non-tumor uptake
Source: PLoS One. 2022 Apr 18;17(4):e0267275. doi: 10.1371/journal.pone.0267275 (PMC9015138; doi:10.1371/journal.pone.0267275)
Supplement: Attachment — Submitted filename: Response to Reviewers_1.docx [file pone.0267275.s003.docx]

Dear Dr. Albano, dear Reviewers,

please find below our response to each point raised by the academic editor and the reviewers.

Journal

1. Please ensure that your manuscript meets PLOS ONE's style requirements, including those for file naming.

The manuscript was revised and should meet now all style requirements of PLOS ONE.

2. Please review your reference list to ensure that it is complete and correct. If you have cited papers that have been retracted, please include the rationale for doing so in the manuscript text, or remove these references and replace them with relevant current references. Any changes to the reference list should be mentioned in the rebuttal letter that accompanies your revised manuscript. If you need to cite a retracted article, indicate the article’s retracted status in the References list and also include a citation and full reference for the retraction notice.

The reference list was reviewed. The cited paper #14 was labeled “ahead of print”. This is incorrect since the paper was published in 2021. We added the publication data.

3. Please provide additional details regarding participant consent. In the ethics statement in the Methods and online submission information, please ensure that you have specified (1) whether consent was informed and (2) what type you obtained (for instance, written or verbal, and if verbal, how it was documented and witnessed). If your study included minors, state whether you obtained consent from parents or guardians. If the need for consent was waived by the ethics committee, please include this information. If you are reporting a retrospective study of medical records or archived samples, please ensure that you have discussed whether all data were fully anonymized before you accessed them and/or whether the IRB or ethics committee waived the requirement for informed consent. If patients provided informed written consent to have data from their medical records used in research, please include this information.

We included the following statement in the Methods section:

“C1 study patients and/or their guardians gave written informed consent and acknowledged transfer and storage of their imaging data on the Pediatric-Hodgkin-Network server [19]. For our evaluation, all PET scans were completely anonymized. Thus, the ethics committee waived the requirement for additional informed consent.”

4. We note that you have provided funding information that is not currently declared in your Funding Statement. However, funding information should not appear in the Acknowledgments section or other areas of your manuscript. We will only publish funding information present in the Funding Statement section of the online submission form. Please remove any funding-related text from the manuscript and let us know how you would like to update your Funding Statement.

We removed the funding-related text from the manuscript. Our funding statement should be:

"The study was supported by the foundation "Mitteldeutsche Kinderkrebsforschung". The funders had no role in study design, data collection and analysis, decision to publish, or preparation of the manuscript."

5. We note that you have included the phrase “data not shown” in your manuscript. Unfortunately, this does not meet our data sharing requirements. PLOS does not permit references to inaccessible data. We require that authors provide all relevant data within the paper, Supporting Information files, or in an acceptable, public repository. Please add a citation to support this phrase or upload the data that corresponds with these findings to a stable repository (such as Figshare or Dryad) and provide and URLs, DOIs, or accession numbers that may be used to access these data. Or, if the data are not a core part of the research being presented in your study, we ask that you remove the phrase that refers to these data.

The data are not a core part of the research being presented in our study. Therefore, we removed the phrase that refers to these data.

Reviewer 1

Why did you only include patients with Hodgkin lymphoma? It seems interesting to add patients with other types of lymphoma mainly DLBCL.

You are right, the classification of patients with non-Hodgkin lymphoma would also be interesting and should be subject of a further study. It would be easily possible to use our method to train different classifiers in a group of DLBCL patients.

Our focus was to develop a classification algorithm for Hodgkin lymphoma patients. Our group is responsible for reference reading of PET scans in the EuroNet - pediatric Hodgkin lymphoma trials. Therefore, our aim is a further improvement of the prognostic information which can be derived from PET. Our next step will be the multifactorial search for prognostic factors for relapse in pretreatment PET of Hodgkin lymphoma patients. The presented automatic classification algorithm is a precondition for this study.

Sensitivity of the algorithm is too low (20% false negative). This is comparable to other algorithms, but hampers the ability to use the algorithm in a real life clinical setting. Please comment on this matter.

It is correct, a sensitivity of 83% probably hampers the use of our classifier in a real life setting, if the algorithm is used alone. However, our automatic algorithm could support the diagnostic work of physicians. Additional information from an automatic classifier will most likely improve the accuracy of the physicians report. Given the complex physiological FDG pattern and the variable occurrence of lymphoma involvement, our results seem to be quite solid, especially since only PET data was used in our study.

Most frequently, false negative lesions occurred in the skeleton and the head-and-neck area. The differentiation between malignant lymph nodes, inflammatory lymph nodes and accessory salivary glands in the neck can be sophisticated, even for experienced physicians. Likewise, the differentiation between physiological increased skeletal uptake and focal skeletal lesions may be also challenging for physicians. In an interreader study of five experts, discrepancies in reporting occurred predominantly in the same areas (*Kluge et al. Inter-Reader Reliability of Early FDG-PET/CT Response Assessment Using the Deauville Scale after 2 Cycles of Intensive Chemotherapy (OEPA) in Hodgkin's Lymphoma. PLoS One. 2016;11:e0149072*). An additional automatic analysis could help to harmonize the reporting.

Specifically, low specificity for gastrointestinal disease might be even more problematic with other types of aggressive lymphoma that tend to involve the GI tract more commonly than Hodgkin lymphoma (eg. DLBCL).

The correct classification of physiological gastrointestinal uptake was an issue in our study. However, none of our study patients had lymphoma involvement of the gastrointestinal tract. Only three patients had mesenterial lymph node lesions. Misclassification occurred between patchy intestinal uptake and abdominal lymph node involvement.

In lymphoma types with a tendency of GI involvement, GI lesions would occur frequently and often with large tumor manifestations in this region. An automatic classifier would probably learn how GI lesions look like and how to discriminate them better from non-malignant GI uptake. Thus, the algorithm performance for the classification of GI-lesions would most likely increase significantly.

Reviewer 2

The articles clearly states that different PET scanners of different centers were used to performe the study. In this setting, more information about the scanner used (PET or PET/CT, analogic or digital scanner, reconstruction algorithm..) are required, better if presented in a table.

We agree, it would be interesting to add an overview of the scanners used. Unfortunately, we are not able to present the data since patient data of all C1 patients were completely anonymized first, for data safety reasons. Thereafter, the 60 patients analyzed in our study were drawn randomly out of all C1 patients. Thus, it is not possible anymore to access and present the scanner data.

The "Automatic Classification" section even if really precise is hard to understand and to follow.

Thank you for this comment. We revised the “Automatic classification” section in the methods part. Now, a simplified explanation of the used method is given here. A detailed description can be found in the supplementary material.

Informations about tumor localization should be given: were all of them nodal localization or other organs were involved?

We added the following information in the “Manual classification” section in the result part:

“Tumor lesions occurred in lymph nodes above and below the diaphragm in 59 and 17 patients, respectively. Extra-nodal lesions occurred in the lung, the skeleton, the liver and the spleen in eight, five, one and ten patients, respectively.”
